# Effects of Different Co-Feeding Protocols on the Early Weaning of Flathead Grey Mullet (*Mugil cephalus*) Larvae

**DOI:** 10.3390/ani13101685

**Published:** 2023-05-18

**Authors:** Raquel Quirós-Pozo, Danilo Concu, Lidia Robaina, Dario Vallainc, Barbara Loi, Javier Roo

**Affiliations:** 1 Grupo de Investigación en Acuicultura, IU-ECOAQUA, Universidad de Las Palmas de Gran Canaria, 35214 Telde, Spain; 2 International Marine Centre—IMC Foundation, Loc. Sa Mardini, Torregrande, 09170 Oristano, Italy; 3 Department of Veterinary Medical Sciences, University of Bologna, 40064 Bologna, Italy

**Keywords:** Mugilidae, rotifers, *Artemia* sp., diversification, gene expression

## Abstract

**Simple Summary:**

Sustainable aquaculture expansion will be crucial for the food security of a growing population expected to reach ~10 billion by 2050. Larval rearing is one of the most complex phases of marine aquaculture due to the need for additional facilities and labor to produce zooplankton to feed the larvae. Therefore, establishing adequate protocols for each species to shift from the live to the inert feed is crucial for more suitable management and profitability of the production. For those reasons, three different co-feeding protocols, A100 (2 initial *Artemia* sp. mL^−1^ day^−1^), A50 (1 initial *Artemia* sp. mL^−1^ day^−1^), and A0 (0 *Artemia* sp. mL^−1^ day^−1^, only rotifers administered as live feed), were evaluated from 22 to 36 days post-hatching (dph) in the weaning of the flathead grey mullet, a promising species for sustainable aquaculture diversification. Survival, growth, biochemical composition, and gene expression of digestive enzymes and growth hormones were assessed. The A0 treatment performed better in survival, while the A100 treatment was the best for growth performance. The expression of the different genes evaluated did not show differences between treatments. In conclusion, rotifers should be maintained until 30–32 dph (total larval length of at least 10 mm) to maximize survival, while *Artemia* sp. supply is recommended from 26 to 29 dph (total larval length of 8 to 9 mm) to improve larval growth and minimize size dispersion.

**Abstract:**

The sustainable expansion of aquaculture relies on a sufficient supply of eggs and larvae, which are the first step of life cycle management. However, marine fish larval rearing generally depends on live feed production, which needs additional facilities and labor. The flathead grey mullet (*Mugil cephalus*), a promising species for aquaculture diversification, has a precocious digestive system development, supporting the feasibility of early weaning strategies. For these reasons, this study evaluated survival, growth, proximate and fatty acid composition, and gene expression of *Mugil cephalus* larvae reared under three different weaning protocols. Three co-feeding treatments, two with different *Artemia* sp. concentrations (A100 and A50, 2 and 1 *Artemia* sp. mL^−1^ day^−1^, respectively) and one with only rotifers administered as live feed along the feeding trial (A0), were assessed from 22 to 36 days post-hatching (dph). The A0 treatment performed better in survival (64.79 ± 7.40%) than the A100 protocol (32.46 ± 12.82%). In contrast, the larvae of the A100 treatment presented significantly higher final length (15.51 ± 0.86 mm) than those of the A0 treatment (12.19 ± 1.45 mm) and higher final weight (41.28 ± 1.48 mg) than those of the A50 and A0 treatments (31.23 ± 3.65 mg and 24.03 ± 7.99 mg, respectively). On the other hand, the expression of digestive enzyme- and somatotropic factor-related genes did not show differences between treatments. The present results support the convenience of treatment A0 in maximizing survival, as rotifers should be maintained until 30–32 dph (until a total larval length of at least 10 mm). However, to improve growth and minimize size dispersion, *Artemia* sp. addition is recommended from day 26 to day 29 post-hatching (total larval length of 8 to 9 mm).

## 1. Introduction

Aquaculture has become the world’s fastest-growing food production sector in the last few decades [1,2], driven by increased demand for aquatic products, overexploitation of wild stocks, and demographic growth. However, one of the most significant bottlenecks for further expansion of the sector is the supply of quality eggs and larvae that can guarantee an adequate volume of juveniles [3].

Larval rearing of marine fish is one of the most expensive and complex phases of the fish production cycle due to the need for auxiliary facilities to produce zooplankton (rotifers (*Brachionus plicatilis* mostly used) and *Artemia* sp.), which in general is critical for the success of the on-growing larval phase and consequently for the rest of the production cycle [4,5]. Many standard larval rearing protocols for Mediterranean species end between 30 and 40 dph [6]; however, the success of early weaning strategies is variable between species. For instance, for the Senegalese sole (*Solea senegalensis*), recent advances have allowed early weaning protocols starting as soon as 15 dph [7], while for the gilthead seabream (*Sparus aurata*), early weaning can begin around 20 dph [8,9]. However, early weaning protocols (16–20 dph) can lead to an increase in mortality or deformity incidence in species such as the European seabass (*Dicentrarchus labrax*) [6,10]. In this context, a suitable weaning protocol may optimize several parameters such as survival, larval growth, and size distribution, directly affecting the subsequent quality of the fingerlings [11]. Therefore, establishing adequate periods and protocols for larval weaning of different species is critical to promoting improved management procedures by hatcheries.

Another measure for the sustainable expansion of aquaculture is species diversification. Among the fish species suitable for this purpose, the Mugilidae family presents great potential due to its euryhaline, eurythermal, and low trophic nature [12]. Nevertheless, its cultivation is still mainly supported by wild fry capture, which results in high mortalities (70% to 96%), and hence appears to be an unsustainable practice [13]. However, the rising interest in mullets in recent years has promoted the investigation of reproductive techniques and physiology in different regions worldwide [14,15,16,17], offering a more sustainable alternative to increase the culture of these species. Specifically, the flathead grey mullet (*Mugil cephalus*) is a great candidate due to its fast growth, its large size, and the high market value of its salted and dried egg roe [18,19].

Recent studies have focused on some aspects of *M. cephalus* larval development under intensive culture conditions, particularly the ontogeny of the digestive, visual, and skeletal systems [19,20]. In addition, the beneficial effects of algal turbidity on larval performance [21] and the effects of different protein levels in weaning diets [22,23] have been recently assessed. Marine larvae such as the flathead grey mullet are born with undeveloped organs and systems that mature during the larval phase to achieve full functionality. For other mullet species such as the thick-lipped grey mullet (*Chelon labrosus*), premature intestinal maturation has been suggested, which may allow the use of early weaning strategies between 14 and 20 days post-hatching [24,25]. In the flathead grey mullet, mouth opening and first intestinal villi formation occur at 3 dph (88 degree days), while gastric glands and other main structures of the digestive tract are developed by 17 dph, which suggests the feasibility of shifting from live to inert feeds from this age onwards [19]. In this context, the expression of the digestive enzyme- and growth factor-related genes in the function of the employed weaning protocol would offer some clues to adapting feeding and rearing conditions [26].

For these reasons, the present study aimed to assess the effects of three different weaning protocols, two with different *Artemia* sp. concentrations (A100 and A50, 2 and 1 *Artemia* sp. mL**^−^**^1^ day**^−1^**, respectively) and one with only rotifers administered as live feed along the feeding trial (A0). Survival, growth performance, proximate and fatty acid composition, and the expression of growth- and digestive enzyme-related genes of *M. cephalus* larvae were evaluated. The present results will provide essential information for tailored management protocols and hatchery cost optimization.

## 2. Materials and Methods

### 2.1. Larval Rearing

Two grey mullet mature females and two mature males were captured during the natural spawning season of the species (September 2020) at the Porto Pino lagoon (Sardinia, Italy), taking advantage of their seasonal migration towards the sea. The animals were transferred to a flow-through 3500 L tank of the San Giuseppe fishing cooperative (50 m from the site of capture) and injected with an LH-RH analog (ENANTONE, Takeda Italia S.p.a) as described by Vallainc et al. [16]. Fertilized eggs were spontaneously obtained 30 h post-injection and transferred to the International Marine Centre Foundation (IMC) facilities in Oristano (Italy). Eggs were incubated in a 2000 L tank in a seawater recirculating aquaculture system (RAS). Ammonia, nitrite, and nitrate levels were monitored and kept below harmful levels (0.5, 1, and 25 mg L^−1^, respectively). After hatching, *M. cephalus* larvae were reared until 14 dph, following a standard protocol for marine larvae [27]. Enriched rotifers (*Brachionus* sp.) were adjusted twice daily to a concentration of 5 rotifers mL^−1^ to achieve a daily dose of 10 rotifers mL^−1^. Two commercial products based on concentrated marine microalgae were used following manufacturer instructions to enrich the rotifers and produce the green water used for the larval rearing (Nanno Star, AlgaSpring, Almere, The Netherlands).

On the fourteenth day post-hatching, larvae were individually counted and manually distributed in nine 300 L tronco-conical tanks in a seawater RAS (1090 larvae per tank, three tanks per treatment), with an initial flow rate of 25% of the tank volume per hour.

Oxygen and temperature were monitored daily with a digital probe (Hach Lange HQ 40 d, Loveland, CO, USA), and the mean recorded values were 8.14 ± 0.10 mg L^−1^ and 23.0 ± 0.16 °C, respectively. Salinity, pH, ammonia, nitrite, and nitrates were measured every 48 h, and the median values were 38.22 ± 0.17 ppt, 7.80 ± 0.03, 0.09 ± 0.10 mg L^−1^, 0.37 ± 0.37 mg L^−1^, and 8.80 ± 5.69 mg L^−1^, respectively. Salinity was measured with a refractometer (ATC Optika S.r.l. Ponteranica, Italy), pH was measured with a pH-meter (Halo HI11312, Hanna Instruments, Padua, Italy), and ammonia, nitrite, and nitrates were measured with a commercial kit (Prodac laboret test kit, Prodac S.L., Citadella, Italy). The bottom of each tank was siphoned daily to remove mortality and uneaten food. The water surface was also cleaned manually every day.

### 2.2. Feeding Protocols

**Figure 1:**
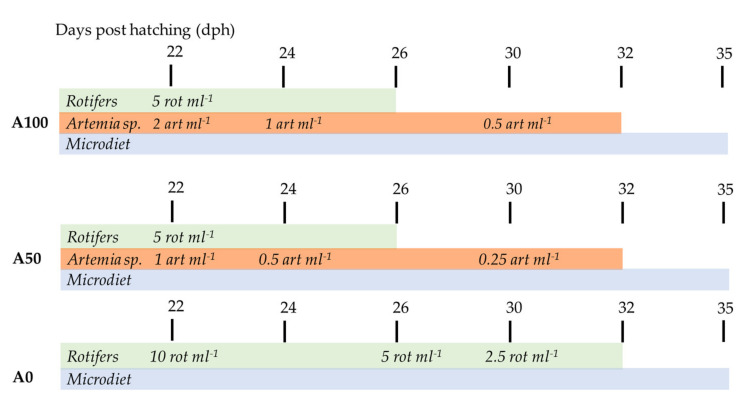
Protocols of daily doses of feed administration along the experiment in the treatments A100, A50, and A0 (2, 1, and 0 initial *Artemia* sp. mL^−1^ day^−1^, respectively).

Larvae were acclimated to experimental tanks for one week, receiving two daily doses of 5 rotifers mL^−1^ in green water culture (*Isocrysis galbana* and *Tetraselmis suecica*, 1:1 in volume ratio in the morning, and Nanno Star green in the afternoon). A 12 h light:12 h darkness artificial photoperiod was used during the whole experiment. Starting from 22 dph, 3 different weaning protocols named A100, A50, and A0 were applied, according to an initial dose of 2, 1, and 0 *Artemia* sp. mL^−1^ day^−1^, respectively (Figure 1). For the control protocol (A100 treatment), initially, rotifers were provided twice daily at a concentration of 2.5 rotifers mL^−1^ (5 rotifers mL^−1^ day^−1^) until 26 dph (end of rotifer administration), and *Artemia* sp. metanauplii were provided four times per day at an initial concentration of 0.5 metanauplii mL^−1^ (2 *Artemia* sp. mL^−1^ day^−1^) with the dose being progressively reduced until 32 dph; thereafter, the larvae were fed only the inert feed until the end of the feeding trial (35 dph). In the A50 treatment, the larvae received the same amount of rotifers (5 rotifers mL^−1^ day^−1^) and a half dose of *Artemia* sp. metanauplii compared to the A100 protocol (four times per day at an initial concentration of 0.25 metanauplii mL^−1^, equal to 1 *Artemia* sp. mL^−1^ day^−1^). In contrast, in the A0 treatment, larvae were fed only rotifers at an initial dose of 10 rotifers mL^−1^ day^−1^ (four daily doses of 2.5 rotifers mL^−1^). In all treatments, the initial doses of live feed were progressively and proportionally decreased, until the end of the live prey feeding period (32 dph) (Figure 1). *Artemia* sp. metanauplii used in all treatments were enriched with the same commercial product used for rotifers, following the manufacturer’s instructions. Furthermore, in all treatments, a commercial microdiet (Gemma micro 0.1 type, Skretting, Vervins, France) (D1) was also offered from the start (22 dph) until the end of the experiment 15 times per day (8:00–18:00 h), and the fed amount was weighed daily. From 31 dph until the end of the feeding period (35 dph), this diet was mixed (1:1) with Gemma wean 0.2 (Skretting, Vervins, France) (D2). From 33 dph onwards, all larvae from the different treatments were fed only with the microdiet. The amount of microdiet was offered considering, on the one hand, the estimated consumption and growth of the larvae to ensure enough feed and, on the other, the visual inspection of the tanks, which allowed the adjustment of the dose given to avoid an excess of uneaten food. The average amount of feed given to each tank was 0.56 g day^−1^ from day 22 to day 29 post-hatching, and 0.88 g day^−1^ from 29 to 35 dph.

### 2.3. Sampling Protocols

The initial sampling was conducted after the acclimatization period (22 dph), prior to the beginning of the experiment. From each tank, 25 larvae were randomly collected and sacrificed with ice-cold water. A subsample of twenty larvae was then measured for total length under a microscope (Leica DMC2900, Wetzlar, Germany) using the LAS 4.5 software (Leica, Wetzlar, Germany). After measurements, a subsample of 15 larvae was placed in 3 groups of 5 larvae on cover glasses previously weighed to determine the wet weight. The glasses were then placed in a laboratory oven at 60 °C for 48 h to determine the dry weight. In addition, 5 larvae were also placed in RNAlater solution, stored at 4 °C for one week, and then frozen at −80 °C until gene expression analyses.

The same procedure was also used to sample larvae at 29 dph (intermediate sampling) and 36 dph (final sampling). At the latter sampling point, a pool of 17 larvae from each tank was also collected for biochemical analyses, frozen at −80 °C, and subsequently lyophilized until analyses could be conducted.

Furthermore, 3 different samples of enriched rotifers and *Artemia* sp. metanauplii were collected, frozen at −80 °C, and lyophilized until biochemical analyses. Samples of the inert diets were also stored at −80 °C until analyses. The biochemical and fatty acid composition of the diets is presented in Table 1.

### 2.4. Survival and Growth

To determine survival, the dead larvae siphoned daily were counted. In addition, at the end of the experiment, the larvae in each tank were counted. Considering the number of larvae sampled during the experimental period, the percentage of survival was calculated using the following formula:

Survival (%) = (final living larvae/(initial larvae at the beginning of the treatments)) × 100

Larval growth was measured using total length and wet and dry weight at the different sampling points. The coefficient of variation of total length (CV) and the specific growth ratio (SGR) were calculated using the following formulas:

CV = (standard deviation of total final length/mean final length) × 100

SGR (%) = (lnWf − lnWi)/days of experiment × 100,

where Wf represents the final weight and Wi represents the initial weight.

### 2.5. Biochemical Analyses

Feed moisture, crude lipid, crude protein, and crude ash content (enriched rotifers, *Artemia* sp. metanauplii, and commercial diets) and larva moisture, crude lipid, and crude protein content were determined in the laboratories of the Instituto Eco-aqua, University of Las Palmas de Gran Canaria (Canary Islands, Spain), following the techniques described by the AOAC [28]. Total crude protein content was determined by the Kjeldahl method [29], and total crude lipid content was determined as described by Folch et al. [30]. The total lipids extracted were trans-esterified [31], and the fatty acids were subsequently quantified by gas chromatography [32]. Analyses of diets were carried out in triplicate, while analyses of larvae were performed in duplicate for each tank (one pool per tank and three pools per treatment).

### 2.6. Gene Expression Analyses

Gene expression analyses were also carried out in the laboratories of the Instituto Eco-aqua, University of Las Palmas de Gran Canaria (Canary Islands, Spain). The present study aimed to assess the expression of genes related to growth (*growth hormone* (*gh*) and *insulin-like growth factor 1* (*igf1*)) and digestive enzymes (*pancreatic alpha amylase* (*amy2a*), *carboxyl ester lipase precursor* (*cel*), *chymotrypsinogen precursor* (*ctr*), *pancreatic phospholipase A2* (*pla2g1b*), and *trypsinogen 2 precursor* (*try2*)). The total RNA of 3 pooled larvae per tank was extracted using TRI reagent (Sigma-Aldrich, Sant Louis, MO, USA) and the extraction kit RNeasy Mini Kit from Qiagen. The reverse transcription (RT) of RNA to cDNA was carried out using the iScript cDNA Synthesis Kit (Bio-Rad Hercules, CA, USA) in 20 μL reaction volume according to the manufacturer’s instructions to be later diluted 1/10 with Milli-Q water. Real-time PCRs were performed using an l-cycler with an optical module (Bio-Rad Hercules, CA, USA) in a final volume of 15.2 μL containing 7.5 μL of iQ-SYBER Green Supermix (Bio-Rad Hercules, CA, USA), 5 μL of cDNA, 0.6 μL of each primer (forward and reverse), and 1.5 μL of Milli-Q water. The conditions for the real-time reactions, which depended on each primer, were adapted from referenced protocols using a gradient of annealing temperatures to determine those with the best efficiency (Table 2). All reactions were performed in duplicate for each sample, and blank control reactions in which the cDNA was replaced with Milli-Q water were included. Relative gene expression was determined by the 2− ΔΔCT method [33] using *18S-rRNA* as a housekeeping gene.

### 2.7. Statistical Analyses

All the statistical analyses were carried out using the program IBM SPSS Statistic 20 (New York, NY, USA). After the normality and homoscedasticity test (*p* ≥ 0.05), the analysis of variance was performed using a one-way ANOVA, and the means were compared using the Tukey post hoc test (*p* ≤ 0.05). When the data did not meet the assumption of normality or homoscedasticity, the medians were compared using a Kruskal–Wallis test (*p* ≤ 0.05).

## 3. Results

### 3.1. Survival and Growth

**Figure 2:**
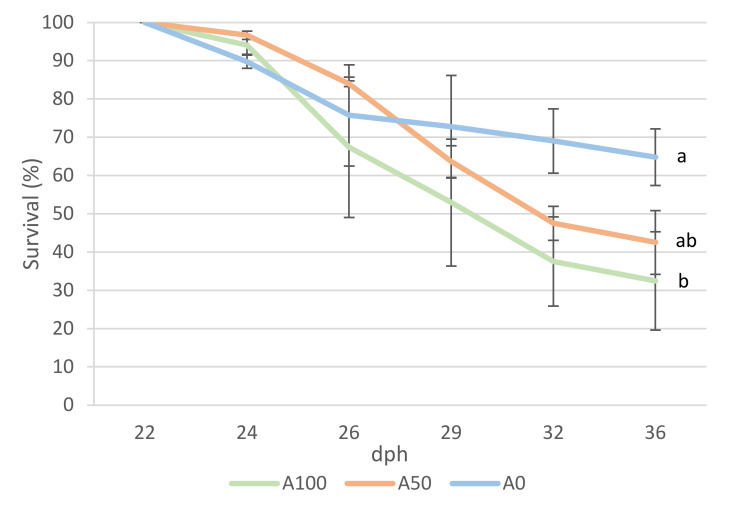
Survival (%) of *Mugil cephalus* larvae fed A100, A50, and A0 protocols (2, 1, and 0 of *Artemia* sp. mL^−1^ day**^−1^**, respectively), during the experiment (means ± SD). Lines with different superscripts indicate the presence of significant differences (Tukey post hoc test *p* ≤ 0.05). Abbreviations: dph, days post-hatching; SD, standard deviation.

The survival of the larvae was significantly different between treatments (*p* = 0.02) (Figure 2). The A100 treatment presented the lowest survival, followed by the A50 treatment, while the A0 treatment resulted in the highest survival.

**Figure 3:**
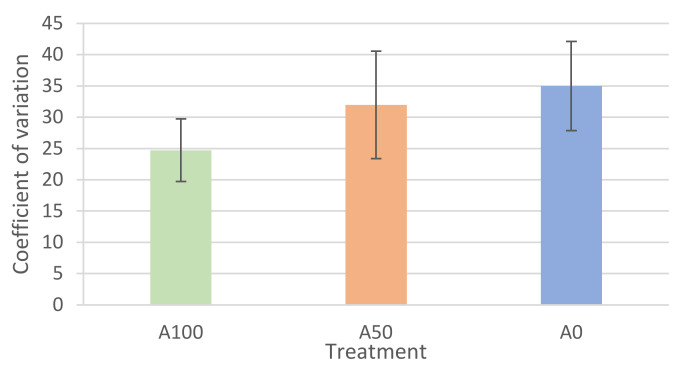
Coefficient of variation (CV) of total length at 36 dph of *Mugil cephalus* larvae fed A100, A50, and A0 protocols (2, 1, and 0 of *Artemia* sp. mL^−1^ day^−1^, respectively) (means ± SD). Columns without superscripts indicate the absence of significant differences (Tukey post hoc test *p* ≥ 0.05). Abbreviations: dph, days post-hatching; SD, standard deviation.

Regarding growth performance, the final length showed differences between treatments; larvae reared under the A100 protocol were significantly longer than those of the A0 treatment at the end of the trial (*p* < 0.01) (Table 3). The CV showed the opposite trend, with the larvae reared under the A0 protocol presenting the highest value, even if not significantly (*p*= 0.27) (Figure 3).

**Figure 4:**
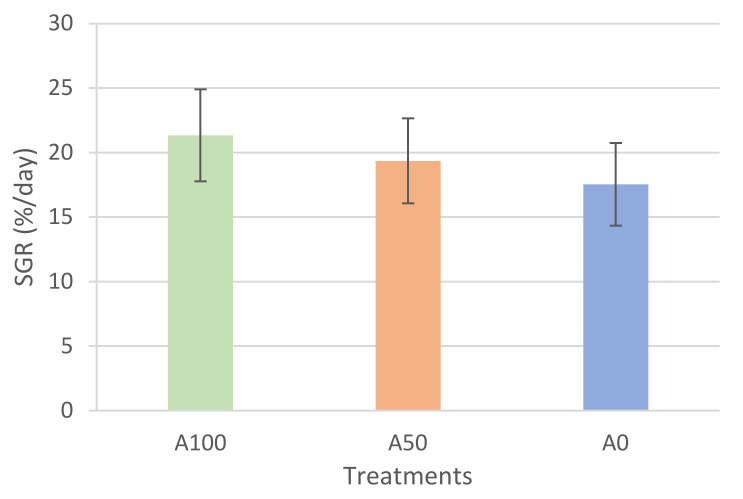
Specific growth rate (SGR (%/day)) at 36 dph of *Mugil cephalus* larvae fed A100, A50, and A0 protocols (2, 1, and 0 of *Artemia* sp. mL^−1^ day**^−1^**, respectively) (means ± SD). Columns without superscripts indicate the absence of significant differences (Tukey post hoc test *p* ≥ 0.05).

Larvae from the A100 treatment also presented a significantly higher wet and dry weight, both at the intermediate and final sampling points (*p* < 0.01) (Table 3). A similar trend was observed for SGR (Figure 4), although the observed differences were not statistically significant (*p* = 0.44).

### 3.2. Biochemical Analyses

The proximate composition of larvae at the end of the experiment (Table 4) showed a similar trend to those observed for survival and final length, as evidenced by the total lipid contents found for the different treatments. The lipid content of the larvae from A100 was significantly higher (*p* = 0.03) than that of larvae under the A0 treatment, while larvae reared with the A50 protocol showed intermediate values. This trend of extreme values for A100 and A0 protocols and intermediate values for A50 was also observed in many cases for the larva fatty acid profile (Table 4). Specifically, the larvae from the A0 treatment presented the highest values of linoleic acid (18:2n-6) (*p* = 0.02), docosapentaenoic acid (DPA) (*p* = 0.03), and DHA/EPA (docosahexaenoic acid/eicosapentaenoic acid) ratio (*p* = 0.02), while the larvae from the A100 and A50 treatment showed the highest levels of oleic acid (18:1n-9) (*p* = 0.01), 18:1n-7 (*p* = 0.01), linolenic acid (18:3n-3) (*p* < 0.01), and n3/n6 ratio (*p* < 0.01).

### 3.3. Gene Expression Analyses

**Figure 5:**
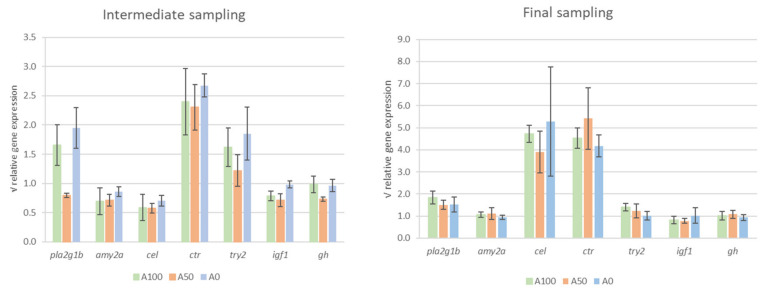
Relative gene expression of digestive enzymes and growth hormones for the different treatments in the intermediate (29 dph) and final sampling (36 dph). Data are normalized by the initial expression rates and are presented as square root of expression ratios ± standard error. Columns without superscripts indicate the absence of significant differences (Tukey post hoc test *p* ≥ 0.05). Abbreviations: *gh*, *growth hormone*; *igf1*, *insulin-like growth factor 1*; *amy2a*, *pancreatic alpha amylase*; *cel*, *carboxyl ester lipase precursor*; *ctr*, *chymotrypsinogen precursor*; *pla2g1b*, *pancreatic phospholipase A2*; *try2*, *trypsinogen 2 precursor*.

**Figure 6:**
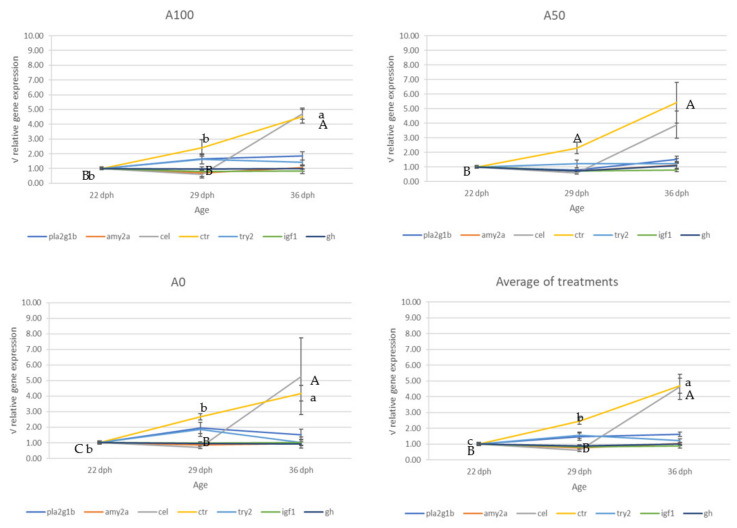
Relative gene expression of digestive enzymes and growth hormones for the different sampling moments. Data are normalized by the initial expression rates and are presented as the square root of normalized expression ratios ± standard error. Different lowercase letters indicate significant differences in *cel* gene expression, while different uppercase letters indicate significant differences in *ctr* gene expression among sampling points (Tukey post hoc test *p* ≤ 0.05). Abbreviations: *gh*, *growth hormone*; *igf1*, *insulin-like growth factor 1*; *amy2a*, *pancreatic alpha amylase*; *cel*, *carboxyl ester lipase precursor*; *ctr*, *chymotrypsinogen precursor*; *pla2g1b*, *pancreatic phospholipase A2*; *try2*, *trypsinogen 2 precursor*.

No differences in the gene expression were found between treatments, neither for the digestive enzyme-related genes nor for the growth hormone-related genes at any of the sampling points (Figure 5). Additionally, the evaluation of the gene expression over time in the different treatments showed that the *carboxyl ester lipase precursor* (*cel*) increased significantly from the intermediate (29 dph) to the final sampling point (36 dph) (*p* = 0.03). In contrast, the *chymotrypsinogen precursor* (*ctr*) increased continuously during the trial (*p* < 0.01) (Figure 6).

## 4. Discussion

Weaning of the flathead grey mullet usually is carried out using a feeding sequence that includes both rotifer and *Artemia* sp. administration until the final shift to inert feeding [15,16,19], as described for other marine aquaculture species. In the present experiment, the live feeding period and the shift to the inert feed were carried out by comparing a standardized protocol (A100) [27] with two protocols, one with reduced *Artemia* sp. administration (A50) and another with no *Artemia* sp. provision (A0). The results evidenced the highest final weight but the lowest survival for the A100 treatment. In contrast, the highest survival and lowest final weight were registered in treatment A0. Survival during the experimental period (Figure 2) remained similar until day 26 post-hatching, the last day of rotifer administration in the A100 and A50 treatments. From that moment onward, mortality continued to increase in the A100 and A50 treatments but not in the A0. This could be related to the relatively small larval mouth size at the time of rotifer removal from the diet which may have prevented an efficient switch from the smaller rotifers to the larger *Artemia* sp. (720 µm average, own data). In this regard, Shirota et al. [36] reported a mouth size between 636 and 882 µm in 5.2 mm *Mugil cephalus* larvae, suggesting an optimum size of the live feed as less than half the size of the mouth gape [36,37]. In our study, at the intermediate sampling point (29 dph), small larvae of around 5.5 mm were still present. Oz et al. [38] also described an important size dispersion in cultured *M. cephalus* larvae and suggested that larval length is a better predictor than age for the larval developmental stage and, therefore, adapting rearing conditions. According to the previous data, this fact may suggest that the smaller larvae in A50 and A100 may have been unable to feed efficiently and cover their nutritional needs when rotifers were removed and *Artemia* sp. became the only live feed available. This may have resulted in higher mortality of the smaller individuals in these treatments and, consequently, larger larval sizes of the survivors.

Additionally, several other factors may have influenced larval survival, particularly when *Artemia* sp. was the primary live feed. For instance, Roo et al. [39] described larval mortality associated with malnutrition in 15–30 dph longfin yellowtail larvae (*Seriola rivoliana*) due to the low digestibility of the chitin exoskeleton of *Artemia* sp., reporting undigested and even alive *Artemia* sp. metanauplii in the feces after their transition thought the larval digestive tract. In addition, the elevated associated bacterial load in *Artemia* sp. (mainly *Vibrio* sp.) has been suggested to cause larval mortality when *Artemia* sp. is the primary food source [40]. Furthermore, present results are consistent with those obtained by Yanes-Roca et al. [41] for pikeperch larvae (*Sander lucioperca*), in which larvae fed only rotifers also presented lower weight and length but higher survival in comparison with those from the treatments where *Artemia* sp. and rotifers were provided. Hence, these authors suggested the higher rotifer concentration as a critical point that increased the chances of the smaller larvae to feed and avoid the point of no return.

The higher weight and length of the larvae in the A100 treatment may have been driven, as discussed above, by the probable higher mortality of the smallest larvae in this treatment and, on the other hand, by the higher lipid content of the *Artemia* sp. and the reduced feeding effort (greater energy intake per capture) which may have also promoted higher growth performance for these larvae. The growth results presented here are in line with previous observations in other species, such as the pikeperch, the Atlantic cod (*Gadus morhua*), and the cobia (*Rachycentron canadum*), in which better growth was obtained for larvae fed a combined protocol of rotifers and *Artemia* sp. in comparison with those fed with only rotifers [41,42,43,44]. Although the results for the CV were not statistically significant, a tendency of greater size dispersion in the larvae from the A0 protocol was observed; this tendency, together with the survival and growth data, supports the hypothesis that the smaller larvae from the A0 protocol were more able to cover their nutritional needs with rotifers and microdiets and therefore achieve a better survival in this treatment.

The higher lipid content of *Artemia* sp. metanauplii compared to rotifers was reflected in the proximate composition of the larvae reared under those protocols, particularly larvae under the A100 treatment. Moreover, the larvae of all treatments presented remarkably higher lipid levels in comparison with those reported for other marine larvae, such as the red porgy (*Pagrus pagrus*) [45], the gilthead seabream [9,46], and the Senegalese sole [46], even when these were fed with equal or even higher lipid content diets compared to those used in the present trial.

The fatty acid profile of the live feed was reflected in the larvae of the different treatments, with higher levels of total n-9, 18:1n-9, 18:1n-7, and 18:3n-3 for the larvae reared under the *Artemia* sp. protocols (A100, A50) and higher levels of 18:2n-6, DPA, EPA and DHA for the larvae reared with rotifers in the A0 protocol, reflecting the fatty acid composition of the prey consumed. Higher DHA and linoleic levels have also been reported for pikeperch larvae reared only with rotifers as live feed compared with those reared with *Artemia* sp. or with a combined protocol [41]. Optimum DHA levels in marine larval feeds range from 0.5 to 2.5% [47], while for *M. cephalus* larvae, Koven et al. (2018, unpublished results) suggested that 1.7% of DHA in the total fatty acids is sufficient in the live feed when a source of 18:3n-3 is provided, which probably may indicate some potential to elongate and desaturate precursors of DHA. In the present study, there were differences in survival between the treatment fed with the higher content of *Artemia* sp. and the treatment fed only with rotifers (with higher highly unsaturated fatty acid (HUFA) content). However, the relationship between dietary DHA and survival seems to be highly species-dependent as it has been demonstrated to have effects on some marine larvae such as the red porgy [45] and the greater amberjack (*Seriola dumerili*) [48], while no effect of this fatty acid in larval survival has been described for other species such as the Senegalese sole [49] and the California halibut (*Paralichthys californicus*) [50]. Galindo et al. [51] concluded that the phylogeny of the fish species might be a relevant factor in the biosynthesis potential of long-chain polyunsaturated fatty acids (LC-PUFAs) from precursors, having demonstrated the ability of both the sand sole (*Pegusa lascaris*) and the thick-lipped grey mullet to synthesize 22:6n-3 from 18:3n-3. These data may suggest a lower compromise of DHA as an essential fatty acid for mullets.

Additionally, the optimal ratios of DHA/EPA for marine larvae have been described to be between 1.2 and 8 [47]. In the present study, the DHA/EPA ratio was lower than these values for rotifers and *Artemia* sp. metanauplii. However, in the larvae, the DHA/EPA ratio was between 2.14 (A100 treatment) and 3.07 (A0 treatment), being comparable to or even higher than those described for other marine larvae [9,45,52] and suggesting a sort of compensatory mechanism for conserving the physiological HUFA ratios, as has been described for other marine species as the Senegalese sole [53] and the gilthead seabream [54].

Regarding gene expression, the different protocols caused no appreciable differences in the digestive enzymes or the growth factors under study. The present findings contrast with those of Koven et al. [21], which suggested the modulation of lipases and alkaline proteases by the diet in *M. cephalus* larvae maintained under different water turbidity conditions. In addition, Koven et al. [23] recorded differential enzyme activities in pancreatic α-amylase, alkaline protease, and trypsin depending on the protein content of the weaning diet. In the present study, the inert weaning diets were the same in all the protocols assayed, which may have masked more subtle changes due to differences in the live feed and may explain the absence of differential expression of the digestive enzymes. Regarding the evaluation of the gene expression during the experiment and irrespectively of the treatment, as in the present study, Gilannejad et al. [26] also recorded a rising trend with time in the gene expression of *ctr* and *cel* in *C. labrosus* larvae. In contrast, they also registered a rising trend in the expression of *pepsinogen 2 precursor*, *pla2g1b*, *gh*, and *ifg1* genes during the same period, which may be explained by the normal ontogenetic variations between the two species or by the different rearing conditions.

The pronounced increase in *cel* and *ctr* observed in our study, mainly from 29 to 36 dph, is indicative of the improved ability of the *Mugil cephalus* larvae at that time to digest both proteins and lipids of the diet. Since in the present experiment, the proximate composition of the two microdiets was quite similar to those of *Artemia* sp. and rotifers, the rise in the expression of both genes may be explained mainly by the intestinal maturation of the larvae during this period, which coincides with the end of the metamorphosis from larvae to the juvenile stage [15]. This hypothesis is also supported by the results of Koven et al. [23], which showed that chymotrypsin and lipase activities of *Mugil cephalus* fry during the weaning phase were independent of the diet provided.

Concerning the gene expression of the somatotropic factors in the different treatments, diverse studies have suggested that poor nutritional status in fish larvae can lead to changes in the expression of the *gh*/*igf* axis [55], indicating that smaller larvae with poorer nutritional status showed higher expression of the growth hormone. Thus, the lack of differences in the expression of the growth-related genes in the present study suggests that the treatments evaluated performed equally in terms of growth potential.

## 5. Conclusions

In conclusion, the present results confirm information on other marine larval weaning protocols and provide new data for a more efficient *Mugil cephalus* weaning. Flathead grey mullet larvae can be successfully weaned directly from rotifers to microdiet by 32 dph with good survival rates. Weaning protocols including artificial diets from 22 dph promoted higher final weight when *Artemia* sp. was provided, while larval survival was reduced. Therefore, to ensure better survival and a smoother transition from live prey to formulated feeds for the smaller larvae, rotifers should be maintained until 30–32 dph (total length of at least 10 mm). Additionally, to minimize size dispersion and to promote a smoother transition between live prey, newly hatched *Artemia* sp. may be provided prior to metanauplii for one to two days; however, *Artemia* sp. metanauplii administration is not recommended until 26–29 dph (total length of 8.0–8.8 mm). The present results highlight the feasibility of applying different weaning strategies for this species and provide hatcheries with new information to optimize the management effort regarding the final costs and benefits during the *Mugil cephalus* weaning phase; however, more studies are needed on this topic for a further understanding of the effects of different weaning protocols on the long-term performance.

## Figures and Tables

**Table 1:** Proximate (% of dry weight) and fatty acid composition (% of total fatty acids (TFAs)) of experimental feeds.

	*Artemia* sp.	Rotifers	D1	D2
**Proximate composition (% dry weight)**				
Lipids	20.11 ± 2.32	16.07 ± 1.13	22.01 ± 1.57	16.20 ± 0.75
Protein	63.66 ± 2.80	62.35 ± 3.58	60.12 ± 0.22	67.97 ± 0.06
Ash	11.55 ± 3.06	12.43 ± 1.20	16.40 ± 0.18	9.08 ± 0.13
**Fatty acid composition (% TFAs)**				
14:0	0.70 ± 0.22	1.29 ± 0.17	1.33	2.44
16:0	14.94 ± 4.67	14.09 ± 1.31	18.4	21.5
16:1n-7	2.81 ± 0.61	9.27 ± 0.36	1.98	3.86
18:0	7.59 ± 2.19	4.61 ± 0.37	4.88	5.48
18:1n-9	23.85 ± 4.35	13.08 ± 2.77	15.85	21.25
18:1n-7	8.33 ± 1.08	4.67 ± 0.21	2.36	2.99
18:2n-6	4.26 ± 1.15	10.71 ± 0.17	29.59	17.07
18:3n-3	21.58 ± 9.95	7.43 ± 0.05	2.67	2.31
20:1n-9	0.06 ± 0.02	0.55 ± 0.13	0.35	0.36
ARA (20:4n-6)	0.74 ± 0.27	3.89 ± 0.70	0.9	0.79
EPA (20:5n-3)	2.13 ± 0.94	5.72 ± 1.68	3.72	3.99
DHA (22:6n-3)	1.30 ± 0.58	3.01 ± 0.71	8.02	6.11
DPA (22:5n-6)	0.27 ± 0.12	1.47 ± 0.36	0.69	0.4
DHA/DPA	4.77 ± 0.03	2.05 ± 0.02	11.59	15.41
ARA/EPA	0.35 ± 0.03	0.69 ± 0.08	0.24	0.2
DHA/EPA	0.74 ± 0.60	0.53 ± 0.03	2.16	1.53
DHA/ARA	2.04 ± 1.53	0.77 ± 0.04	8.9	7.76
Oleic/DHA	6.09 ± 1.06	1.59 ± 0.50	1.98	3.48
Oleic/n-3 PUFAs	0.92 ± 0.53	0.63 ± 0.22	1.01	1.5
n-3/n-6	4.73 ± 0.92	1.09 ± 0.05	0.49	0.75
Total SFAs	24.00 ± 6.87	20.56 ± 1.93	25.29	30.38
Total MUFAs	38.17 ± 6.83	34.93 ± 2.94	26.3	35.09
Total n-3	29.60 ± 12.30	22.75 ± 2.86	15.94	14.51
Total n-6	6.12 ± 1.41	20.79 ± 1.67	32.58	19.32
Total n-9	24.39 ± 4.35	15.85 ± 1.94	16.68	22.18
Total n-3 PUFAs	29.51 ± 12.32	21.45 ± 3.23	15.76	14.17
Total n-6 PUFAs	1.28 ± 0.22	7.71 ± 1.48	2.08	1.67

Data expressed as means ± SD. Contains: 14:1n-7, 15:1n-5, 16:2n-6, 16:2n-4, 16:3n-4, 16:3n-3, 16:3n-1, 16:4n-3, 16:4n-1, 18:1n-5, 18:2n-4, 18:3n-6, 18:3n-4, 18:3n-1, 18:4n-1, 20:0, 20:1n-5, 20:2n-9, 20:2n-6, 20:3n-9, 20:3n-6, 22:1n-11, 22:1n-9, and 22:4n-6. Abbreviations: ARA, arachidonic acid; D1, microdiet Gemma micro 0.1 type, Skretting; D2, microdiet Gemma wean 0.2 type, Skretting; DHA, docosahexaenoic acid; DPA, docosapentaenoic acid; EPA, eicosapentaenoic acid; MUFAs, monounsaturated fatty acids; PUFAs, polyunsaturated fatty acids; SD, standard deviation; SFAs, saturated fatty acids.

**Table 2:** Primer sequences and RT-PCR conditions of the different genes analyzed.

Gene	Access. Number	Primer Sequence 5′–3′		Initial Denaturation (°C) (Duration in min)	Denaturing Temperature	Annealing Temperature (°C) (Duration in s)	Extension Temperature (°C) (Duration in s)	Number of Cycles	Reference
					(°C) (Duration in s)				
*pla2g1b*	MH350433	F	ACACCTGTTGATGACCTGGA	95 (3)	95 (15)	58.5 (30)	58.5 (30)	35	[26]
		R	GTCTTGGTGGCCTTGTCAC	95 (3)	95 (15)		58.5 (30)	35	
*amy2a*	KF684941	F	CCAAACTGGGAACTGTCATCAG	95 (3)	95 (15)	58.5 (30)	58.5 (30)	35	[26]
		R	TCTGGTTGTCGTGGTTGTCA	95 (3)	95 (15)		58.5 (30)	35	
*cel*	MH350432	F	CTGACCATGCTGATGACCTG	95 (3)	95 (15)	60 (30)	58.5 (30)	35	[26]
		R	GGCAATCATGTAACCGGAGA	95 (3)	95 (15)		58.5 (30)	35	
*ctr*	KC195969	F	CGTCCCTTCAGGATTATACCG	95 (3)	95 (15)	60 (30)	58.5 (30)	35	[26]
		R	AGTTGGAGGAACGGTCATGTT	95 (3)	95 (15)		58.5 (30)	35	
*try2*	KF684940	F	CTCCAGAACACAGCCATGAAG	95 (3)	95 (15)	60 (30)	58.5 (30)	35	[26]
		R	ACGTTCAGAGAGGCCTGGTAG						
*igf1*	AY427954.1	F	TCT TCA AGA GTG CGA TGT GC	95 (3)	95 (15)	53.8 (30)	72 (30)	35	[34]
		R	ACA GCT TTG GAA GCA GCA CT						
*gh*	AF134605.1	F	CATG CAC AAG GTG AGG AAG A	95 (3)	95 (15)	53.8 (30)	72 (30)	35	[34]
		R	AGG TCT CAA CCT GCA AAC ATC						
*18S-rRNA*		F	CACATCCAAGGAAGGCAGCA	94 (2)	94 (30)	60 (30)	68 (30)	30	[35]
		R	AAGATACGCTATTGGAGCTG						

Abbreviations: *gh, growth hormone; igf1, insulin-like growth factor 1; amy2a, pancreatic alpha amylase; cel, carboxyl ester lipase precursor; ctr, chymotrypsinogen precursor; pla2g1b, pancreatic phospholipase A2; RT-PCR, real-time polymerase chain reaction; try2, trypsinogen 2 precursor*.

**Table 3:** Wet weight (WW), dry weight (DW), and total length (TL) of *Mugil cephalus* larvae reared under the different protocols, during the experiment.

	Initial (22 dph)			Intermediate (29 dph)			Final (36 dph)		
	WW (mg)	DW (mg)	TL (mm)	WW (mg)	DW (mg)	TL (mm)	WW (mg)	DW (mg)	TL (mm)
A100	1.82 ± 0.80	0.46 ± 0.09	5.60 ± 0.16	6.31 ± 1.33 ^a^	1.83 ± 0.27 ^a^	8.79 ± 0.33	41.28 ± 1.48 ^a^	9.84 ± 0.26 ^a^	15.51 ± 0.86 ^a^
A50	1.79 ± 0.64	0.42 ± 0.06	5.56 ± 0.08	3.78 ± 1.16 ^b^	1.09 ± 0.21 ^b^	8.03 ± 0.12	31.23 ± 3.65 ^b^	7.23 ± 1.02 ^b^	14.01 ± 1.24 ^ab^
A0	1.74 ± 0.69	0.46 ± 0.08	5.73 ± 0.27	3.95 ± 1.61 ^b^	1.15 ± 0.23 ^b^	8.01 ± 0.75	24.03 ± 7.99 ^b^	5.31 ± 2.02 ^b^	12.19 ± 1.45 ^b^

Data expressed as means ± SD. Values of the means in the same column with different superscripts indicate the presence of significant differences (Tukey post hoc test *p* ≤ 0.05). Abbreviations: dph, days post-hatching; SD, standard deviation.

**Table 4:** Proximate (% of dry weight) and fatty acid composition (% of total fatty acids (TFAs)) of the whole larvae from the different experimental groups.

	A100	A50	A0
**Proximate composition (% dry weight)**			
Lipids	25.49 ± 0.73 ^a^	23.42 ± 0.86 ^ab^	23.17 ± 1.01 ^b^
Protein	66.82 ± 1.78	69.72 ± 2.28	69.46 ± 2.28
**Fatty acid composition (% TFAs)**			
14:0	1.43 ± 0.05	1.31 ± 0.15	1.43 ± 0.32
16:0	21.61 ± 1.69	20.52 ± 1.41	21.41 ± 2.66
16:1n-7	3.62 ± 0.16	3.32 ± 0.42	3.86 ± 0.46
18:0	7.76 ± 0.73	7.92 ± 0.20	7.98 ± 0.51
18:1n-9	22.42 ± 0.91 ^a^	21.04 ± 1.02 ^ab^	19.18 ± 0.80 ^b^
18:1n-7	6.20 ± 0.46 ^a^	5.54 ± 0.33 ^a^	4.36 ± 0.10 ^b^
18:2n-6	8.67 ± 1.09 ^b^	10.00 ± 0.72 ^ab^	11.50 ± 0.79 ^a^
18:3n-3	6.32 ± 0.98 ^a^	5.49 ± 0.27 ^a^	2.82 ± 0.24 ^b^
20:1n-9	0.30 ± 0.02 ^b^	0.34 ± 0.03 ^b^	0.42 ± 0.02 ^a^
ARA (20:4n-6)	1.05 ± 0.08	1.21 ± 0.19	1.45 ± 0.21
EPA (20:5n-3)	2.51 ± 0.41	2.86 ± 0.41	2.99 ± 0.39
DHA (22:6n-3)	5.37 ± 1.10	7.38 ± 1.73	9.18 ± 1.94
DPA (22:5n-6)	0.37 ± 0.06 ^b^	0.50 ± 0.11 ^ab^	0.69 ± 0.15 ^a^
DHA/DPA	14.46 ± 0.88 ^b^	14.81 ± 0.28 ^ab^	13.26 ± 0.55 ^a^
ARA/EPA	0.42 ± 0.05	0.42 ± 0.01	0.49 ± 0.06
DHA/EPA	2.14 ± 0.11 ^b^	2.58 ± 0.24 ^ab^	3.07 ± 0.38 ^a^
DHA/ARA	5.09 ± 0.72	6.07 ± 0.46	6.32 ± 0.69
Oleic/DHA	4.33 ± 1.13	2.97 ± 0.74	2.17 ± 0.60
Oleic/n-3 PUFAs	1.30 ± 0.30	1.11 ± 0.16	1.07 ± 0.22
n-3/n-6	1.56 ± 0.12 ^a^	1.47 ± 0.06 ^a^	1.18 ± 0.09 ^b^
Total SFAs	31.50 ± 2.50	30.49 ± 1.52	31.57 ± 3.27
Total MUFAs	37.51 ± 1.71	35.48 ± 1.52	33.35 ± 0.97
Total n-3	17.88 ± 2.97	19.46 ± 2.12	18.49 ± 2.98
Total n-6	11.43 ± 1.29 ^b^	13.23 ± 1.14 ^ab^	15.65 ± 1.37 ^a^
Total n-9	23.76 ± 0.87 ^a^	22.31 ± 1.05 ^ab^	20.58 ± 0.73 ^b^
Total n-3 PUFAs	17.64 ± 2.96	19.20 ± 2.11	18.26 ± 2.98
Total n-6 PUFAs	11.06 ± 1.23 ^b^	12.74 ± 1.04 ^ab^	14.96 ± 1.23 ^a^

Data expressed as means ± SD. Values of the means in the same row with different superscripts indicate the presence of significant differences (Tukey post hoc test *p* ≤ 0.05). Contains: 14:1n-7, 15:1n-5, 16:2n-6, 16:2n-4, 16:3n-4, 16:3n-3, 16:3n-1, 16:4n-3, 16:4n-1, 18:1n-5, 18:2n-4, 18:3n-6, 18:3n-4, 18:3n-1, 18:4n-1, 20:0, 20:1n-5, 20:2n-9, 20:2n-6, 20:3n-9, 20:3n-6, 22:1n-11, 22:1n-9, and 22:4n-6. Abbreviations: ARA, arachidonic acid; DHA, docosahexaenoic acid; DPA, docosapentaenoic acid; EPA, eicosapentaenoic acid; MUFAs, monounsaturated fatty acids; PUFAs, polyunsaturated fatty acids; SD, standard deviation; SFAs, saturated fatty acids.

## Data Availability

Not applicable.
